# Common Perceptions of Periodontal Health and Illness among Adults: A Qualitative Study

**DOI:** 10.5402/2012/671879

**Published:** 2012-09-17

**Authors:** M. Gholami, A. Pakdaman, J. I. Virtanen

**Affiliations:** ^1^Department of Community Oral Health, School of Dentistry, Tehran University of Medical Sciences, P.O. Box 1439955991, Tehran, Iran; ^2^Department of Oral Public Health, Institute of Dentistry, University of Helsinki, P.O. Box 41, 00014 Helsinki, Finland; ^3^Department of Community Dentistry, Institute of Dentistry, University of Oulu, P.O. Box 5281, 90014 Oulu, Finland; ^4^Department of Public Health, University of Helsinki, P.O. Box 41, 00014 Helsinki, Finland

## Abstract

*Objective*. Our aim was to explore perceptions of periodontal health and illness and to examine attitudes and beliefs regarding prevention of gum diseases among Iranian adults. *Methods*. Our qualitative approach included focus-group discussions among adults aged 18 and above based on convenient and purposive sampling in Tehran. Transcripts of the four focus-group discussions were analyzed by two independent reviewers using a content analysis method. *Results*. Two major themes in the analyses emerged: the common perception of periodontal health and illness and the attitude towards prevention. The study demonstrated the subjects' good understanding of prevention of periodontal disease, but their lack of knowledge of the aetiology of the diseases, and an inability to differentiate aetiology, symptoms, and prevention of dental caries and periodontal disease. *Conclusion*. This study revealed a need for oral health education among Iranian adults to improve their knowledge and change their attitudes to achieve deeper understanding of the aetiology and prevention of periodontal disease. Health promotion programs should address misconceptions about prevention of gum disease.

## 1. Introduction

Periodontal disease is one of the two main and most prevalent oral diseases all over the world. The disease affects subjects in all age groups but is more common in adult populations [1, 2]. In addition, oral hygiene, diet, smoking, diabetes mellitus, male gender, and various socioeconomic factors are considered among the risk factors for periodontal disease [3–7]. Periodontitis is often preceded by gingivitis, which in the early stages of the disease process causes inflammation and bleeding during tooth brushing. If not properly treated, it often proceeds to periodontitis with destruction of connective tissues and finally results in tooth loss.

The situation regarding periodontal disease varies among regions and countries [1]. WHO data show that periodontal diseases are common and comprise a serious health problem, for example, in many developing countries [8]. In the Eastern Mediterranean countries, the periodontal health status measured by the Community Periodontal Index (CPI) in 35- to 44-year-old showed that about 90% of individuals had signs of the disease varying from gum bleeding to deep periodontal pockets [9]. Tooth loss ascribed to severe periodontitis has commonly been apparent in 5% to 15% of adult populations, and in Eastern Mediterranean countries, this figure has been between 7% and 46% in older people [10, 11]. 

A national survey of the prevalence of periodontal disease among adults aged 35 to 44 with CPI scores of two and above, signifying detection of calculus during probing and pocket depths of 4 mm and more, was discovered in more than 80% of the Iranian population in 2002 [12]. While these findings clearly indicate impaired periodontal health status among adults, there is also lack of information on the perception of periodontal health and illness among the adult population.

Because periodontal diseases often progress slowly, without clear signs and symptoms, especially in their early stages, awareness of and the proper attitude toward these diseases are crucial for health promotion and disease prevention. To gain information on views and values related to periodontal health and illness and possible effects of the particular culture requires a qualitative prior to a quantitative approach. Thus, the purpose of the present study was to explore perceptions of periodontal health and illness and to examine attitudes and beliefs regarding prevention of gum diseases among Iranian adults.

## 2. Materials and Methods

A qualitative study including focus-group discussions provided in-depth understanding of individual and group experiences and perception. Participants were aged 18 years and over and resided in Tehran. Four focus-group discussions, described in detail in Table 1, with lay people were organized based on purposive sampling to reflect the diversity of socioeconomic level. To access the adult population, an announcement went to four public schools: two boys' and two girls' schools, chosen from low- and high-socioeconomic regions of Tehran. These provided volunteer parents, selected by age and gender, who were invited to take part in the group discussions.

One experienced researcher as moderator, together with an assistant, conducted focus groups in which the moderator asked participants to express themselves and urged them to share their experiences freely. The moderator presented guiding questions in the form of open-ended questions including three main concepts: the main oral health problems, periodontal health and disease, and perceived means of prevention. The moderator explained all the essential facts regarding discussions. Each session lasted, on average, for about one hour. Participants received small gifts at the end of the session. To establish dependability; the sessions were tape recorded and transcribed.

### 2.1. Ethics

The study was voluntary, and participants received information orally about the study aims. At the beginning of the discussion, all participants provided their verbal consent. The research was approved by the Tehran University of Medical Science ethics committee.

## 3. Statistical Analysis

Transcript analysis was performed by two independent reviewers (M.G., A.P.) using a “thematic analysis” method. The analyses consisted of three stages; firstly, transcripts were reviewed several times, and each meaning unit was extracted as a code. In case of disagreement in coding, differences were resolved via discussion. Similar codes for meaning were then grouped and condensed into categories considered as higher level headings. This process continued, in order to extract themes. Agreement between reviewers occurred by a process of reflection and discussion as to how to identify and sort the codes according to their similarities and differences, in line with Graneheim & Lundman [13]. Data gathering continued during focus groups to reach saturation, that is, new information no longer emerges.

## 4. Results

The demographic characteristics of the focus-group participants are presented in Table 1. In total, 46 individuals participated.

The two major themes emerging in relation to the purpose of the study were “common perception of periodontal health and illness” and “attitude towards prevention”, which are presented in Table 2.

Participants considered oral health to play an important role in relation to general health and believed the same attention should be paid to oral health problems as to other general health problems. They mentioned a relationship between oral health and heart and gastrointestinal health, appropriate chewing, and good social interactions. Among oral diseases, they cited periodontal disease as of high priority. Furthermore, the participants placed a higher value on gingival health than on dental health because of the vital role of the gums in tooth retention. 

### 4.1. Common Perceptions of Periodontal Health and Illness

#### 4.1.1. Perceived Aetiology

They addressed the lack of oral hygiene and vigorous tooth brushing which causes trauma to the gums as being aetiologic factors for gum disease. The participants recognized that consumption of sugar in addition to lack of vitamins causes gum disease. Several participants mentioned a causative relationship between tooth decay and gum disease and even mentioned development of gum disease when tooth discolouration occurs.

*When a tooth is decayed and becomes yellow and black, it can affect one's gums (a woman aged 35+).*

Some inevitable factors such as aging and hereditary tooth structure were considered to be aetiological factors.

*My teeth became loose because of ageing, and I've got spaces between my teeth resulting in food impaction. I try to remove food debris by all accessible means such as with a strand of plastic or a thread, only causing injury and infection of my gums (a man aged 35+).*

*In my opinion, hereditary factors and tooth “tissue” are very important. My husband never pays attention to his oral hygiene, he smokes too and doesn't use dental floss, but he still has healthy teeth and gums. In comparison, even though I take care of my teeth and gums more than he does, most of my teeth have been restored with crowns (a young woman).*

#### 4.1.2. Perceived Symptoms

Moreover, in relation to periodontal disease, the participants mentioned discoloration, swelling, pain, recession and infection of gums, tooth loss, and tooth abscess as important symptoms of periodontal disease. However, spontaneous gum bleeding or bleeding with gum stimulation was considered more important. They also mentioned aphthous ulcers as another manifestation of gum disease which might worry them.

### 4.2. Attitude towards Prevention

#### 4.2.1. Self-Care

In order to prevent periodontal disease, participants described the importance of maintaining good oral hygiene by means of tooth brushing, dental flossing, and rinsing with mouthwash or salt water. Dental floss was considered a tool to remove impacted food debris.

*Regular and correct tooth brushing, three times a day or at least once at night, is necessary to prevent gum disease (a young man).*

*When food debris such as meat pieces sticks between the teeth, we use dental floss to remove it (a young woman).*

#### 4.2.2. Healthy Diet

Some participants highlighted the importance of a healthy diet, in particular consumption of dairy products and vitamin C, as well as fruits and vegetables. 

#### 4.2.3. Regular Check-Up

The participants considered regular dental check-ups as important; however, further discussions revealed that they usually do not follow this behaviour due to lack of time, laziness, busy life style, cost, and lack of dental insurance.

*Usually we go to visit a dentist when we face oral problems such as pain. Dentists recommend a regular dental visit every 4 to 6 months, but we cannot follow that due to several daily problems (a young woman).*

#### 4.2.4. Social Values

The respondents commonly reported the impact of oral health on aesthetics and social interactions. They mentioned the significance of appearance and mouth odours especially in maintaining good family relationship as very important. Young women complained that this is neglected. It was emphasized in the discussions that aesthetics is considered as a major and effective factor in communication, which can increase individuals' self-confidence.

*Regarding the individuals with bad oral odour, it's clear they consider it as of no value for themselves and other people who have to tolerate them (a young woman).*

Moreover, participants mentioned the importance of appearance as a stimulating factor to act in a preventive manner such as in brushing their teeth and visiting a dentist. With regard to periodontal health, aesthetics was considered the most important indication for scaling to improve the appearance of the gums and to remove tooth stains.

*Children pay more attention to aesthetics when they grow up. My daughter has noticeably large teeth, and when I mentioned it to her, she decided to brush them regularly (a young woman).*

#### 4.2.5. Cultural Traditional Aspects

(1) Traditional Beliefs. These participants expressed some cultural traditional beliefs related to the prevention of gum problems. They believed not consuming cold and hot foods simultaneously prevents gum disease.

*We should make a delay between drinking of hot tea and cold water because this intermittent consumption of cold and hot foods can cause gum and tooth problems (a man aged 35+).*

Rubbing the teeth with coal was believed to be a good method to maintain oral hygiene, and also eating hard fruits and vegetables such as apple and carrot instead of tooth brushing was mentioned as acceptable to clean the teeth.

*When we forget brushing teeth, for example when travelling, we can eat an apple or carrot before bed, which can clean the teeth sufficiently (a woman aged 35+).*

(2) Application of Home Remedies. Home remedies reported to improve gingival problems included using baking soda dissolved in water for gargling to prevent gum infection or boiled sumac to relieve gum problems.

*I use a herbaceous drug. I boil sumac and gargle its extract and rub it on my gums. It has a great effect in relieving bleeding and swelling of gums (a women aged 35+).*

A majority of the participants had a positive view toward rinsing the mouth with salt water to cure gingival problems, that is, gum bleeding or looseness.

## 5. Discussion

Our study showed an overall favourable attitude toward and appreciation of periodontal health and fairly good knowledge with regard to prevention of periodontal disease among the participants. While the most important preventive methods such as good oral hygiene, diet, and regular dental visits were acknowledged, the participants showed a low level of knowledge of the aetiology of periodontal disease. In particular, they failed to clearly differentiate between the aetiology, symptoms, and prevention of dental caries and periodontal disease. 

Based on their viewpoints and beliefs about periodontal health and illness, two major themes emerged in this study: “common perception of periodontal health and illness” and “attitude towards prevention”.

### 5.1. Perception

The participants generally held the view that poor oral hygiene is the cause of periodontal disease, but they were unaware of the relevant role of dental plaque. Most of the participants knew regular tooth brushing to be the main method of maintaining good oral hygiene, and some of them referred to the use of dental floss only to remove food debris, similarly as found in a national study [14] which reported the use of matches instead of dental floss. Hereditary weak gum/tooth structure was considered as a predisposing factor for periodontal disease. The results show that there is confusion between perceptions of healthy gums and healthy teeth among lay people. Any noticeable change in the mouth including aphtous ulcer was considered a sign of gum disease. Apparently they had other misconceptions involving predisposing factors and their perception of periodontal health and illness, misconceptions about the effect of sugar and tooth decay on gum disease. Even gum disease was considered as a consequence of discoloured teeth.

Even though the participants chose to visit a dentist mainly only when symptoms occurred, a finding common in many societies [15, 16], they agreed that regular dental visits could be effective in preventing periodontal disease, in contrast to findings among elderly and adult groups of Chinese [15].

### 5.2. Social Importance

One of the most influential factors regarding the importance of periodontal health was related to aesthetics and external appearance, which have been important, for example among adolescents [17]. In addition, the participants even highlighted aesthetics as a stimulative factor, making them seek oral health care [18, 19].

### 5.3. Cultural Aspects

Certain cultural traditional beliefs related to periodontal health and disease were brought to light. The mixing cold and hot food as an aetiological factor of periodontal disease is a belief among the Chinese [20]. Traditional home remedies to relieve oral and periodontal problems have been common, for instance African-Americans using cotton balls soaked in an aspirin solution or alcohol to relieve pain and swelling [21]. Among the Chinese, drinking “cooling tea” and gum massage with alum or musk are common methods to treat gum disease [22, 23]. Similarly, the participants in our study used home remedies such as baking soda and boiled sumac to relieve periodontal problems. Various species of sumac have served indigenous cultures for medicinal purposes, such as the native people of North America, who have traditionally used *Rhusglabra* (smooth sumac) to treat bacterial diseases such as syphilis, gonorrhea, and gangrene [24]. In the Mediterranean countries and the Middle East, *Rhus coriaria* (tanner's sumac) commonly serves as a medicinal herb, particularly for wound healing [25]. Application of salt dissolved in water was, in our study, also among the traditional means to cure gum pain, swelling, bleeding, and looseness. Similar practices have been reported elsewhere, for instance in America and Asia [18, 22, 26]. Among mothers and teachers in rural Qazvin, use of highly concentrated salted water was effective in making gums firm [14].

### 5.4. Methodological Aspects

Our qualitative approach utilizing in-depth investigations allowed us to gain a deeper understanding of individuals' beliefs and attitudes regarding periodontal diseases. In addition, we took into account the socioeconomic backgrounds in focus groups purposive sampling. In order to provide a supportive and stimulating atmosphere for discussions in the focus groups, we invited appropriate groups of participants, considering age and gender as important factors, a method used in qualitative investigations [14]; men, however, were not as cooperative as the women, perhaps due to their being busy at work at the time of group discussions. This study utilized the method of tape recording and transcribing the sessions for establishing dependability of the data. Two independent reviewers carried out the analysis process to extract themes after agreement achievement.

In focus groups, the moderator strove to keep the discussion circulating, especially when someone dominated; in all focus groups; however, the discussion continued until general agreement was achieved. Keeping in mind the nature of the study method, our findings bring to light the views and perceptions of adults; especially regarding age groups other than those studied here, the results should thus be interpreted with caution. 

## 6. Conclusion

This qualitative study provides useful information on perceptions of periodontal health and illness as well as attitudes towards prevention of gum disease among Iranian adults. Our findings indicate a substantial need for oral health education to improve their knowledge and change their attitudes, especially to achieve deeper understanding of the development and prevention of periodontal disease. The two major themes uncovered can serve in future promotion of oral health at the community level.

## Figures and Tables

**Table 1:** Characteristics of the participants in focus-group discussions.

	Focus group 1:	Focus group 2:	Focus group 3:	Focus group 4:
	older women	young women	young men	older men
	(*n* = 22)	(*n* = 12)	(*n* = 6)	(*n* = 6)
	(41, 32–47)*	(35, 30–39)	(41, 37–44)	(49, 42–54)
Gender				
Male			6	6
Female	22	12		
Socioeconomic area				
Low	22		6	
High		12		6
Education				
Primary/secondary	9	4	2	
High school/Dipl.	13	8		2
University			4	4

*Mean age, range.

**Table 2:** Categories and themes identified from the focus-group discussions.

Themes	Categories
Common perceptions of periodontal	Perceived aetiology
health and illness	Perceived symptoms

	Self-care
	Healthy diet
Attitude towards prevention	Regular check-up
	Social values
	Cultural traditional aspects
